# Psyllium fiber improves hangovers and inflammatory liver injury by inhibiting intestinal drinking

**DOI:** 10.3389/fphar.2024.1378653

**Published:** 2024-06-28

**Authors:** Keungmo Yang, Tom Ryu, Beom Sun Chung

**Affiliations:** ^1^ Division of Gastroenterology and Hepatology, Department of Internal Medicine, College of Medicine, The Catholic University of Korea, Seoul, Republic of Korea; ^2^ Department of Internal Medicine, Institute for Digestive Research, Digestive Disease Center, Soonchunhyang University College of Medicine, Seoul, Republic of Korea; ^3^ Department of Anatomy, Yonsei University Wonju College of Medicine, Wonju, Republic of Korea

**Keywords:** psyllium fiber, hangover, alcohol-induced liver injury, inflammation, alcohol absorption

## Abstract

**Introduction:** Excessive alcohol intake often results in hangovers and inflammatory liver damage, posing a significant health concern. Current treatment options for hangovers are still insufficient, highlighting the urgent need for new therapeutic approaches. Psyllium fiber (PF) is well-known for its gastrointestinal benefits, but its effect on hangovers is less explored.

**Methods:** We utilized a mouse model with a single binge drinking (4 g/kg) to induce hangover and inflammatory liver injury. Intestine and liver injury were serologically and histologically estimated. Hangover symptoms were assessed using cylinder and footprint tests to objectively quantify hangover symptoms in mice.

**Results:** Binge drinking significantly activated alcohol-metabolizing enzymes in the small intestine and liver, leading to inflammatory damage. Concurrently, there was a rise in alcohol metabolites such as acetaldehyde and acetone, which exhibited a positive correlation with hangover symptoms in mice. Interestingly, the oral administration of PF (100 mg/kg) alongside alcohol consumption significantly reduced the activity of these enzymes and lowered the levels of alcohol metabolites. Mice treated with PF exhibited a considerable improvement in hangover symptoms and a reduction in hepatic inflammation, compared to control groups. Furthermore, *in vitro* experiments using HepG2 cell lines and semipermeable membranes demonstrated that PF effectively inhibits alcohol absorption into the body.

**Discussion:** In conclusion, PF demonstrates a potential protective effect against alcohol-induced hangover and liver injury by inhibiting the absorption of alcohol and lowering hangover-related alcohol metabolites. This study suggests that PF could serve as an effective therapeutic option for mitigating the adverse effects of excessive alcohol consumption.

## Graphical Abstract

**Figure:**
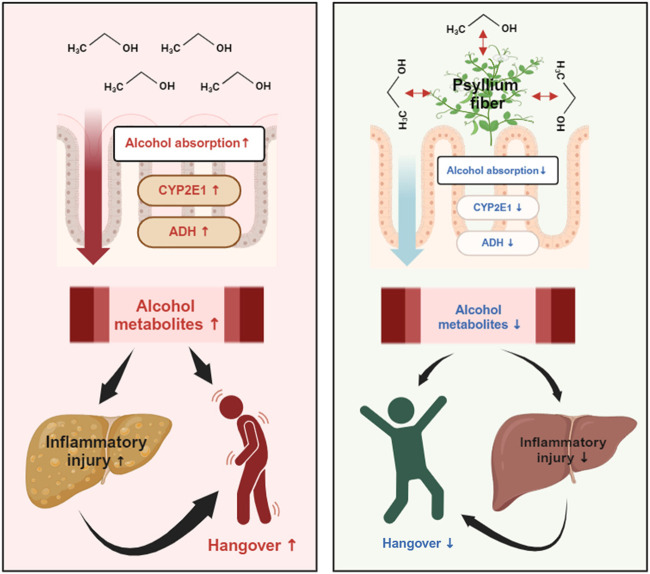
Graphical summary of the study.

## 1 Introduction

In recent years, the significant rise in chronic alcohol consumption has emerged as a major global health concern, contributing to a spectrum of alcohol-associated liver diseases including steatosis, steatohepatitis, cirrhosis, and hepatocellular carcinoma (Kim et al., 2020). Meanwhile, in the regard of acute alcohol consumption, people suffer from acute intoxication called hangover, which is characterized by an unpleasant feeling and symptoms, and it is first described by the British writer William Hickey in 1,768 as an aftermath of heavy alcohol drinking (Swift and Davidson, 1998). Although the symptoms of hangover include distress, fatigue, thirst, palpitation, tremor, sickness, diarrhea, and headache, they have interindividual variability and affect functional and psychiatric problems (Prat et al., 2009). Amount of alcohol costs is $7.5 billion per year, and the diminished occupational productivity caused by hangover-like symptoms lost $1.4 billion each year in Canada (Single et al., 1998). More than 1 million workdays are deleted per year due to the symptoms of hangover in Finland (Järvilehto et al., 1975). Moreover, the majority of alcohol-related workplace issues are attributed to light to moderate drinkers, with hangover being the primary morbidity rather than long-term consequences of chronic alcohol consumption, such as alcohol-associated liver diseases (Stockwell, 1998).

Physiologic mechanisms of alcohol hangover include direct effects of alcohol, and alcohol metabolism such as acetaldehyde toxicity (Swift and Davidson, 1998). Alcohol-induced diuresis by inhibiting antidiuretic hormone or vasopressin, and that leads the body to electrolyte imbalance and dehydration (Eisenhofer et al., 1985). Additionally, alcohol itself can irritate the gastrointestinal wall and disrupt the normal gut barrier, resulting in upper abdominal pain, nausea, and vomiting (Lieber, 1995). Furthermore, vasodilation related headache could be occurred with alcohol intoxication and alteration of secretion of several neurotransmitters with alcoholic effect would be implicated in the pathogenesis of headache (Parantainen, 1983).

Alcohol is metabolized by two-step process. The first enzyme that is called alcohol dehydrogenase (ADH) changes alcohol to an acetaldehyde, then second enzyme called aldehyde dehydrogenase (ALDH) metabolizes acetaldehyde to acetic acid. The speed of conversions is controlled by the concentration of nicotinamide adenine dinucleotide. Furthermore, excessive alcohol breakdown requires cytochrome P 450 2E1 (CYP2E1) and co-factor nicotinamide adenine dinucleotide phosphate (Mackus et al., 2020). Among them, acetaldehyde, an intermediate product of alcohol metabolism, is known to causes toxic effects, including tachycardia, skin flushing, nausea and headache. Interestingly, in some people genetic variants with the ALDH enzyme release become ill after consuming small amounts of alcohol (Swift and Davidson, 1998; Brooks and Zakhari, 2014). Furthermore, alcohol withdrawal, the presence of congeners, concurrent use of other drugs, and individual differences may exacerbate hangover symptoms.

Although interventions for hangover symptoms are searched with several results in the internet, including aspirin, bananas, exercise, hair of the dog, ibuprofen, shower, and sleep, effective way to relieve hangover was not studied scientifically so far. Recently, several interesting phytomedical interventions were introduced for decrease hangover feeling, and that included Borago officinalis, Cynara scolymus, Opuntia ficus-indica (Pittler et al., 2003; Wiese et al., 2004; Pittler et al., 2005). Nevertheless, all the compounds had insignificant efficacy for hangover relief or was not designed as a strictly controlled study (Pittler et al., 2005). Psyllium fiber consists of gel-forming arabinoxylan, a polymer rich arabinose, which is rarely digested in human gastrointestinal tract (Jalanka et al., 2019). Psyllium fiber is well-known laxatives that have capacity for effective water holding and prevent from dehydration of bowel (McRorie Jr and McKeown, 2017). This gel-forming fiber is also studied as an intervention for obesity and diabetes (Gibb et al., 2015; Gibb et al., 2023).

The study proposes that psyllium fiber, known for its hydrophilic properties and gel-forming ability, could be an effective remedy for hangovers. This hypothesis is based on the idea that psyllium fiber can influence alcohol absorption in the intestines through osmosis. Given these properties, we aim to investigate whether psyllium fiber can alleviate hangover symptoms and alcohol-induced liver injury by modulating alcohol absorption in the gut.

## 2 Methods

### 2.1 Psyllium fiber

In this experiment, a widely recognized commercial product (Whole Psyllium Husks, NOW Foods, Inc., Bloomingdale, IL) within its expiration date was utilized. This product contains psyllium fiber sourced from the seeds of Plantago ovata Forssk [Plantaginaceae]. The selection of this product was based on its extensive use and established reliability, ensuring the consistency and reproducibility of the experimental results.

### 2.2 Animal experiment

The Institute Animal Care and Use Committee (IACUC) in Yonsei University Wonju College of Medicine approved all animal experiments (IACUC number: YWC-230614-2). The mice were managed on the regular light-dark cycle (12 h each) in a specific pathogen-free (SPF) facility. The SPF facility’s temperature and humidity were consistently maintained at around 22°C ± 2°C and 40%–60%, respectively. Wild-type (WT) mice with C57BL/6J background were purchased from Orient Bio Inc. (Seongnam, Republic of Korea). Single binge drinking of ethanol (4 g/kg body weight) was performed to induce hangovers and inflammatory liver injury in the 8-week-old male mice. To assess the effects on hangovers, the mice were either given a vehicle or psyllium fiber (100 mg/kg) by oral gavage together with the binge drinking. The study grouped 24 mice into four different categories, with each group consisting of 6 mice per cage.

### 2.3 Cell culture

Human hepatocellular carcinoma cells (HepG2) were purchased from the ATCC (Manassas, VA). The standard medium used was Dulbecco’s Modified Eagle Medium (DMEM), supplemented with 10% fetal bovine serum (FBS) and antibiotics (penicillin 10,000 units/mL, streptomycin 10,000 μg/mL). The cells were cultured in 6-well plates at a density of approximately 200,000 cells per well in 2 mL of standard medium for *in vitro* experiments. This particular seeding density was chosen to ensure adequate cell-to-cell contact and optimal cell growth, while avoiding over-confluence that could lead to cellular stress and altered cellular responses. HepG2 cells were incubated at 37°C in a humidified atmosphere of 5% CO_2_ to ensure optimal growth conditions.

### 2.4 Biochemical measurement

The mice were sacrificed after 12 h of the binge drinking, and their venous blood samples were collected in vacuum tubes containing ethylenediaminetetraacetic acid anticoagulant (V-tube, AB Medical, Republic of Korea) and centrifuged at 14,000 rpm for 5 min. The supernatant was analyzed using gas chromatography on an Agilent 7890 B Gas Chromatograph (Agilent Technologies, Santa Clara, CA, United States). The levels of alanine aminotransferase (ALT), aspartate aminotransferase (AST), triglycerides (TG), and total cholesterol (TC) were analyzed using Fuji Dry-Chem 3,500 (Fuji Film, Tokyo, Japan).

### 2.5 Quantitative PCR

Total RNA was isolated from either liver tissues or cells using either the RNeasy Mini Kit (Qiagen) or the TRIzol reagent (Thermo Fisher Scientific), following the manufacturer’s instructions. This RNA was then uniformly reverse-transcribed into cDNA using the ReverTra Ace^®^ qPCR RT Master Mix with gDNA Remover (Toyobo). For the quantitative real-time PCR (qRT-PCR), the SYBR Green Real-time PCR Master Mix (Toyobo), was employed in with Quantstudio 3 (PCR Instrument system; Thermo Fisher Scientific). To standardize the relative expression levels of each interested genes, the expression level of 18S rRNA was utilized as a reference. The detailed information of the forward and reverse primer pairs used are presented in Supplementary Table S1.

### 2.6 Histological analyses

For uniformity in histological analysis across all samples, the same areas of the medial and left liver lobes as well as the jejunum area of the intestine were consistently collected from each mouse. The tissue samples were then fixed in 10% neutral buffered formalin (Sigma-Aldrich) for an overnight period. Following a thorough deparaffinization and rehydration process, the tissues were sectioned into 4 μm slices and subsequently stained using hematoxylin (Sigma-Aldrich) and eosin (Biognost) solutions. Imaging of the stained tissues was performed using an Olympus BX51 light microscope, and the DP2-BSW software was employed for the analysis of the captured images.

### 2.7 Immunostaining

Paraffin-embedded liver tissue sections from mice were used for immunostaining analysis. The process began with deparaffinization and rehydration of the samples, followed by their immersion in a 10 mM citrate buffer (pH 6.0). For antigen retrieval, the samples underwent microwave exposure for 5 min. To prevent non-specific binding, the tissues were treated with 10% donkey serum for an hour. Overnight incubation at 4°C was then carried out with primary antibodies diluted between 1:50 to 1:200 in PBS containing 0.1% Tween-20. For immunohistochemistry staining, the samples were incubated with either anti-Rabbit IgG (Vector Laboratories) or anti-Mouse IgG (Vector Laboratories) for an hour at room temperature. The DAB substrate kit (Vector Laboratories) was used to develop the further reactions, and the slides were sealed with Balsam (Sigma-Aldrich). In the case of immunofluorescent staining, the samples were treated with Alexa Fluor^®^ 488 or Alexa Fluor^®^ 594 conjugated secondary antibodies (Abcam) for an hour at room temperature and then covered with DAPI mounting solution (Abcam). The imaging of these samples was carried out using an Olympus BX51 microscope (Olympus, Tokyo, Japan). For image analysis, DP2-BSW software was utilized.

### 2.8 Western blot analyses

Proteins were extracted from tissues using RIPA lysis buffer, which includes 10% glycerol, 10% SDS, 1 mM Na_3_VO_4_, 1 mM PMSF, 150 mM NaCl, and 30 mM Tris (pH 7.5), along with phosphatase inhibitor and protease cocktail (Thermo Fisher Scientific). These proteins were then separated using 10% SDS-polyacrylamide gel electrophoresis. For transferring the proteins from the gel, a nitrocellulose membrane (Thermo Fisher Scientific) was utilized. After the transfer, this nitrocellulose membrane was subjected to blocking with a 5% skim milk solution for an hour at room temperature. Following this, the membranes were treated overnight at 4°C with primary antibodies diluted in PBS (1:500 to 1:2,000). On the next day, the membranes were incubated with secondary antibodies for 1 h at room temperature. The SuperSignal™ West Pico PLUS Chemiluminescent substrate (Thermo Fisher Scientific) was applied to visualize the immunoreactive bands.

### 2.9 Isolation of liver mononuclear cells

The liver tissues of the mice were homogenized using a 70 μm mesh filter cell strainer. This is followed by centrifugation at 40 g for 5 min to remove hepatocytes. The supernatant obtained is then washed and resuspended in 40% Percoll (GE Healthcare). This cell lysate was subsequently centrifuged at 1,400 g and 4°C for 30 min to further purify the sample. Red blood cells were lysed using an RBC lysis buffer (BioLegend). The isolated liver mononuclear cells (MNCs) were then washed in sterile PBS and their numbers were quantified.

### 2.10 Flow cytometry analyses

The liver mononuclear cells (MNCs) isolated from mice were initially stained with CD16/CD32 (mouse Fc blocker) (BD Bioscience) and then with the LIVE/DEAD™ fixable aqua dead cell stain kit for 405 nm excitation (Thermo Fisher Scientific) to differentiate between living and dead cells. Subsequently, these cells were labeled with various fluorescence-tagged antibodies: eFluor 450-conjugated CD45 (Thermo Fisher Scientific), APC-Cy7-conjugated CD11b (BD Biosciences), FITC-conjugated F4/80 (eBioscience), PE-Cy7-conjugated Ly-6G (BD Biosciences), and PE-conjugated Siglec-F (BD Biosciences). The fluorescence-labeled cells were then analyzed using a FACS Aria III flow cytometer (BD Bioscience). Data regarding the proportions of neutrophils (CD11b^+^Ly6G^+^), eosinophils (CD11b^+^SiglecF^+^), and macrophages (F4/80^+^CD11b^+^) were examined using FlowJo software (FlowJo LLC) through a pseudo-color analysis plot.

### 2.11 Semipermeable membrane experiment

To simulate alcohol diffusion through the cell membrane, a semipermeable membrane experiment was designed. The semipermeable membrane (Innovating Science) was composed of regenerated cellulose derived from cotton linters, which are among the purest naturally occurring sources of cellulose. This membrane permits the passage of particles up to 14,000 Da. Ethanol, with a molecular weight of approximately 46 Da, was able to diffuse through the semipermeable membrane, and its concentration was measured over time. The semipermeable pocket contained 50 mL of normal saline with 5 g of psyllium fiber.

### 2.12 Statistical analysis

All collected data were analyzed using Prism software, version 8.0 (GraphPad Software), and are represented as mean ± SEM. In the *in vivo* experiments, any mice that experienced severe problems, such as mortality post-binge drinking, were excluded in further analysis. Statistical differences between two groups were determined using the unpaired Student’s *t*-test. For comparisons involving multiple groups, One-way Analysis of Variance (ANOVA) was employed, along with *post hoc* tests like Tukey’s and Dunnett’s. A *p*-value of less than 0.05 was considered statistically significant.

## 3 Results

### 3.1 Binge drinking activates alcohol metabolism in intestines

**FIGURE 1:**
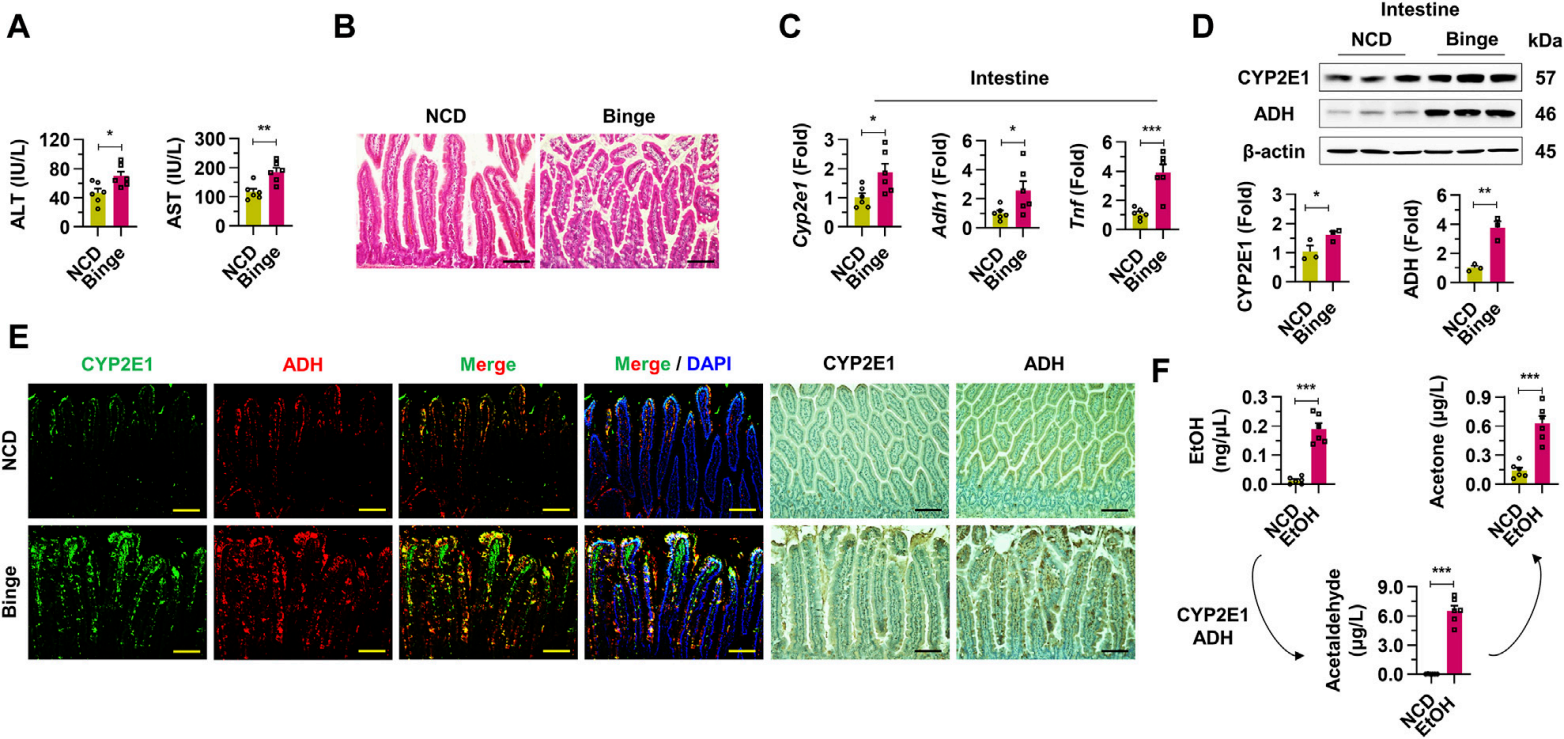
Binge drinking activates intestinal alcohol metabolism through upregulation of cytochrome P450 2E1 and alcohol dehydrogenases in mice. **(A)** Serum alanine aminotransferase (ALT) and aspartate aminotransferase (AST) were investigated from normal chow diet (NCD) and binge drinking groups (n = 6/group). **(B)** Representative H & E staining pictures from intestine samples. Scale bar, 50 μm. **(C)** Relative mRNA expressions of *Cyp2e1*, *Adh1*, and *Tnf* were estimated in intestine tissues (n = 6/group). **(D)** Western blot analysis of CYP2E1 and ADH in intestine tissues. **(E)** Representative immunofluorescence and immunohistochemistry staining pictures of CYP2E1 and ADH from intestine sections. Scale bar, 50 μm. **(F)** Ethanol (EtOH), acetaldehyde, and acetone levels were evaluated from whole blood in mice with binge drinking (n = 6/group). Data are presented as mean ± SEM. *p < 0.05, **p < 0.01, ***p < 0.001.

In this research, we employed a binge drinking (4 g/kg body weight) model to induce hangover symptoms and alcohol-induced liver damage in mice. Compared to the normal chow diet (NCD) group, the binge drinking mice exhibited significantly elevated serum ALT and AST levels, suggesting liver damage (Figure 1A). H&E staining demonstrated epithelial cell damage and steatosis in the intestinal tissues of the binge group relative to the NCD group (Figure 1B). The mRNA expressions of Cyp2e1, Adh1, and Tnf were markedly upregulated in the intestines of the binge group (Figure 1C). Protein levels of CYP2E1 and ADH showed a substantial increase in the intestines of the binge-exposed mice, as confirmed by Western blot analysis (Figure 1D). Similarly, enhanced expression of CYP2E1 and ADH in the intestines of the binge drinking mice was evident through immunofluorescence and immunohistochemistry staining (Figure 1E). Furthermore, elevated levels of ethanol, acetaldehyde, and acetone in the blood provided further evidence of activated alcohol metabolism following binge drinking (Figure 1F). These findings collectively indicate that binge drinking significantly induces damage to intestinal cells and activates alcohol metabolism in the intestines.

### 3.2 Inflammatory liver injury was induced by binge drinking

**FIGURE 2:**
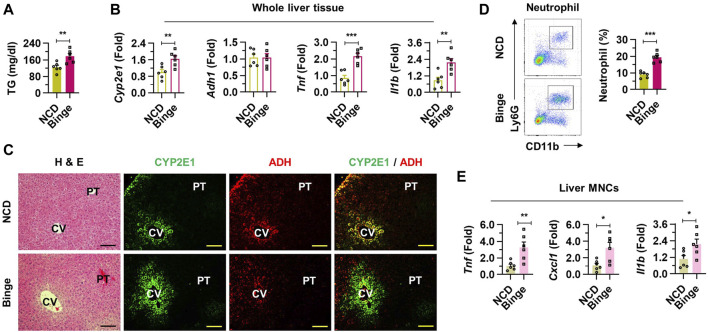
Inflammatory liver injury was induced by binge drinking in mice. **(A)** Serum triglyceride (TG) levels were measured after binge drinking (n = 6/group). **(B)** Relative mRNA expressions of *Cyp2e1*, *Adh1*, *Tnf*, and *Il1b* were analyzed in whole liver tissues (n = 6/group). **(C)** Representative H & E and immunofluorescence (CYP2E1 and ADH) staining pictures in liver sections. Central vein (CV) and portal triad (PT). Scale bar, 50 μm. **(D)** Frequencies of neutrophils from isolated liver mononuclear cells (MNCs) were estimated by fluorescence activated cell sorting. **(E)** qRT-PCR of *Tnf, Cxcl1*, and *Il1b* in liver MNCs (n = 6/group). Data are presented as mean ± SEM. *p < 0.05, **p < 0.01, ***p < 0.001.

There were no significant changes in total body weight and liver to body weight ratio observed between two groups (Supplementary Figure S1A). Elevated serum triglyceride (TG) levels were observed in the binge drinking mice, while total cholesterol (TC) levels showed no significant difference when compared to the NCD group (Figure 2A; Supplementary Figure S1B). mRNA expressions of Cyp2e1, Adh1, Tnf, and Il1b were significantly upregulated in the whole liver tissues of the binge group, indicating enhanced metabolic activity (Figure 2B). Interestingly, the number of liver mononuclear cells (MNCs) significantly increased after alcohol consumption (Supplementary Figure S1C). Consistently, with alcohol administration, there was evidence of hepatocyte damage and mild steatosis around the central veins (CV) of the liver, along with activation of enzymes associated with alcohol metabolism (Figure 2C; Supplementary Figure S1D). An increased frequency of neutrophils from hepatic MNCs in the binge group indicated an inflammatory response, while the frequency of macrophages and eosinophils showed no significant change (Figure 2D; Supplementary Figure S1E). qRT-PCR analysis further confirmed the inflammatory state with elevated expression levels of Tnf, Cxcl1, and Il1b in liver MNCs (Figure 2E). Taken together, these results underline the specific pathological and inflammatory effects of binge alcohol consumption on liver tissue.

### 3.3 Alcohol metabolites are associated with hangover symptoms in mice

**FIGURE 3:**
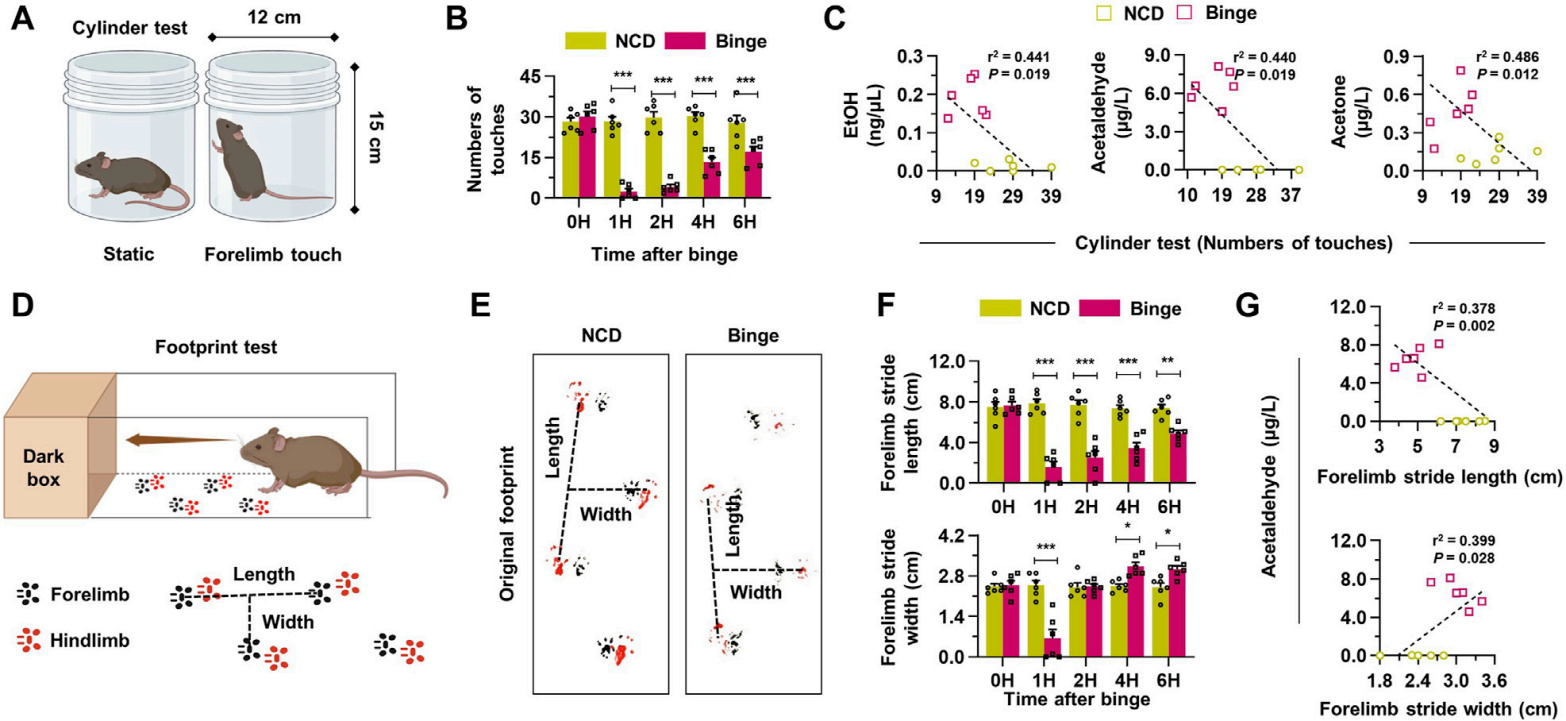
Binge drinking elevates the production of alcohol metabolites, leading to hangover behaviors. **(A)** A schematic figure of cylinder test (static position or forelimb touch) in mice. **(B)** Binge drinking leads to a decrease in the number of touches in the cylinder test at various time points (n = 6/group). **(C)** Correlative analysis was conducted to assess the relationship between alcohol metabolites and the results of the cylinder tests in both the NCD and binge-drinking groups (n = 6/group). **(D)** A diagram illustrating the footprint test in mice (black: forelimb, red: hindlimb). **(E)** Representative pictures of the footprint test taken 6 h after binge drinking. **(F)** Forelimb stride length and width according to the different time points after binge drinking (n = 6/group). **(G)** Correlative analysis between footprint tests and blood acetaldehyde levels (n = 6/group). Data are presented as mean ± SEM. *p < 0.05, **p < 0.01, ***p < 0.001.

In the assessment of the impact of binge drinking on behavior and metabolism, we utilized various tests. The cylinder test depicted in Figure 3A involves two positions: static and forelimb touch. Binge drinking led to a decrease in the number of touches in the cylinder test over various time points, suggesting impaired motor function (Figure 3B). Correlative analysis showed a significant negative correlation between increased alcohol metabolites and decreased motor activity in the cylinder test results (Figure 3C). The footprint test serves as another quantitative approach for objectively measuring hangover symptoms by analyzing gait changes, captured through the tracking of movements in the forelimb and hindlimb (Figure 3D). Six hours after binge drinking, the footprint test revealed specific alterations in the gait of mice, such as a reduced stride length and an increased stride width, mimicking the staggered gait commonly observed in individuals under the influence of alcohol (Figure 3E). A significant correlation was noted between forelimb stride lengths and blood alcohol metabolite levels, showing a negative relationship, while stride width displayed a positive correlation with metabolite levels, indicating significant changes in gait patterns linked to increased alcohol metabolites (Figure 3F; Supplementary Figure S2A). Similarly, the analysis of hindlimb strides also indicated results consistent with those of the forelimb, further demonstrating the motor impairments induced by binge drinking (Supplementary Figure S2C). Thus, binge drinking in mice effectively resulted in impaired motor function and altered gait patterns, mimicking hangover symptoms, which were associated with increased alcohol metabolites.

### 3.4 Psyllium fiber inhibits alcohol absorption in intestine

**FIGURE 4:**
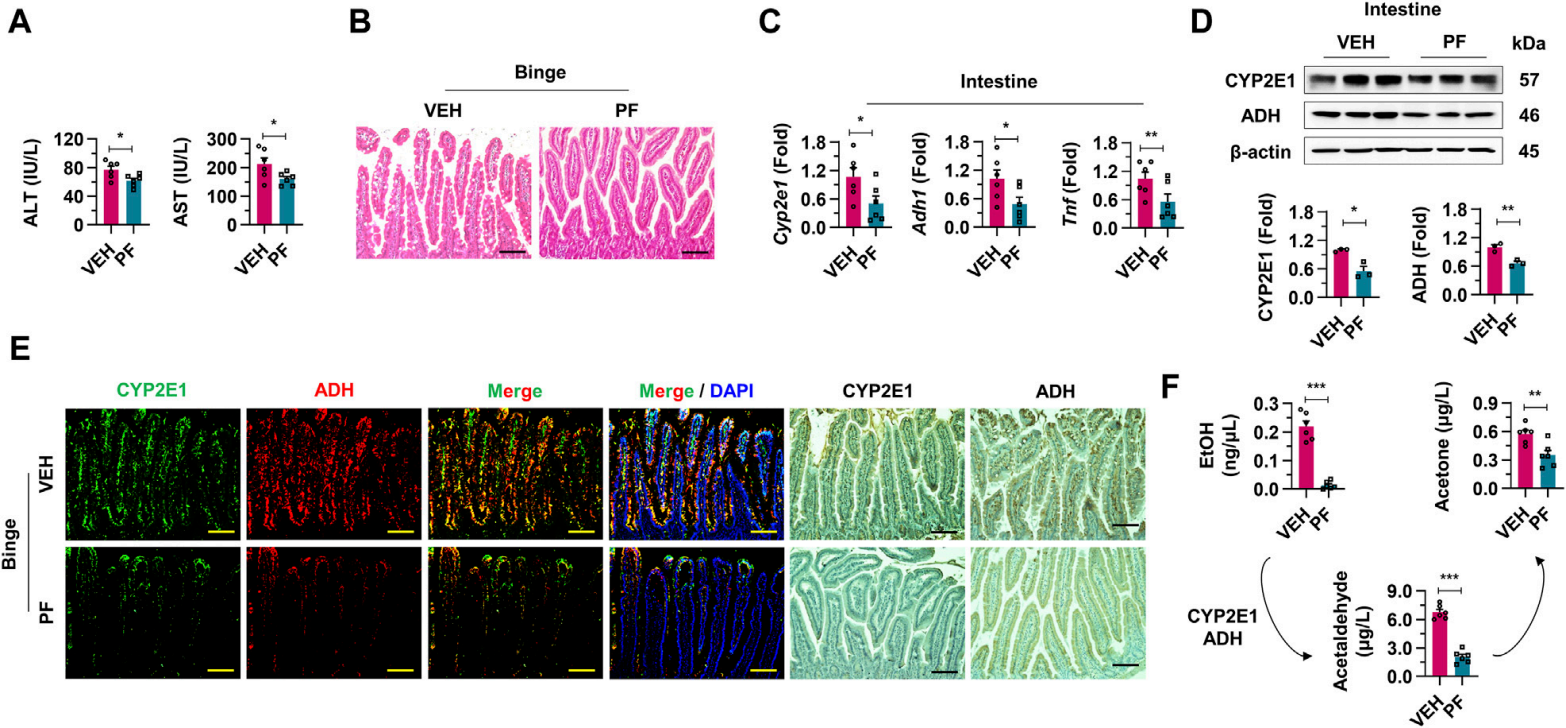
Psyllium fiber inhibits alcohol absorption in intestine. **(A)** Serum ALT and AST levels were analyzed in vehicle (VEH) or psyllium fiber (PF)-treated mice with binge drinking (n = 6/group). **(B)** Representative H & E staining pictures in intestine sections. Scale bar, 50 μm. **(C)** qRT-PCR of *Cyp2e1*, *Adh1*, and *Tnf* in intestine tissues (n = 6/group). **(D)** Western blot analysis of CYP2E1 and ADH in the mice intestine tissues. **(E)** Immunofluorescence and immunohistochemistry staining pictures of CYP2E1 and ADH in mice treated with VEH or PF. Scale bar, 50 μm. **(F)** Blood ethanol (EtOH), acetaldehyde, and acetone levels were analyzed in VEH or PF-treated mice with binge drinking (n = 6/group). Data are presented as mean ± SEM. *p < 0.05, **p < 0.01, ***p < 0.001.

To evaluate the efficacy of psyllium fiber in modulating the effects of alcohol absorption, mice subjected to binge drinking were administered with vehicle or psyllium fiber (Figure 4). First, a significant reduction in both AST and ALT levels was observed in the group treated with psyllium fiber compared to the vehicle group (Figure 4A). After evaluating serological inflammatory markers, psyllium fiber was found to reduce alcohol-induced damage to intestinal epithelial cells and steatosis. These findings underscore the histopathological benefits of psyllium fiber in preserving the integrity of the intestinal mucosa upon alcohol exposure (Figure 4B). In qRT-PCR and Western blot analyses, mice treated with psyllium fiber displayed a notable decrease in mRNA and protein expression levels of CYP2E1 and ADH compared to the vehicle-treated group. This reduction indicates that PF may exert a regulatory effect on the metabolic pathways engaged in alcohol processing in the intestine (Figures 4C, D). Immunostaining analysis also showed diminished localization and expression of CYP2E1 and ADH enzymes in the intestinal tissues of psyllium fiber-treated mice, aligning with the qRT-PCR and Western blot data (Figure 4E). Lastly, psyllium fiber treatment remarkably decreased the blood levels of ethanol, acetaldehyde, and acetate, indicating that psyllium fiber effectively reduced alcohol absorption in the intestine (Figure 4F).

### 3.5 Alcohol-induced hepatic inflammation is attenuated after psyllium fiber treatment

**FIGURE 5:**
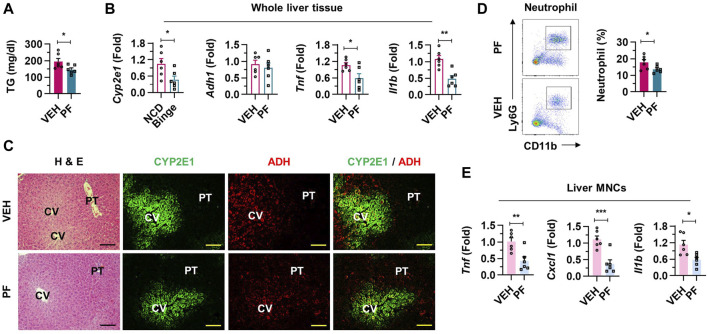
Alcohol-induced inflammatory liver injury is attenuated in psyllium fiber-treated mice. **(A)** Serum triglycerides (TG) levels were analyzed in vehicle (VEH) or psyllium fiber (PF)-treated mice after binge drinking (n = 6/group). **(B)** Relative mRNA expressions of *Cyp2e1*, *Adh1*, *Tnf*, and *Il1b* were estimated in whole liver tissues (n = 6/group). **(C)** Representative H & E and immunofluorescence staining (CYP2E1 and ADH) pictures in liver sections. Central vein (CV) and portal triad (PT). Scale bar, 50 μm. **(D)** Frequencies of neutrophils from isolated liver mononuclear cells (MNCs) calculated by FACS. **(E)** qRT-PCR of *Tnf*, *Cxcl1*, and *Il1b* from isolated liver MNCs (n = 6/group). Data are presented as mean ± SEM. *p < 0.05, **p < 0.01, ***p < 0.001.

Next, we investigated the impact of psyllium fiber on hepatic inflammation induced by binge drinking. Psyllium fiber treatment did not significantly affect total body weight or the liver to body weight ratio (Supplementary Figure S3A). There was a notable reduction of serum TG levels without the alteration of TC levels (Figure 5A; Supplementary Figure S3B). Psyllium fiber treatment resulted in the downregulation of pro-inflammatory genes such as Cyp2e1, Adh1, Tnf, and Il1b, indicating inhibited alcohol-metabolizing activity and inflammatory signaling in the liver tissues (Figure 5B). This decrease in the number of hepatic MNCs is consistent with the anti-inflammatory effects observed at the molecular level (Supplementary Figure S3C). Histological analysis revealed reduced damage in hepatocytes and decreased steatosis around the CVs in the psyllium fiber-treated mice. Furthermore, a decline in the expression of alcohol-metabolizing enzymes CYP2E1 and ADH was observed (Figure 5C; Supplementary Figure S3D). Flow cytometry analysis of isolated liver MNCs from the psyllium fiber-treated group showed a significant reduction in neutrophil frequency, without notable changes in the counts of macrophages and eosinophils, further supporting the anti-inflammatory properties of psyllium fiber in alcohol-induced liver injury (Figure 5D; Supplementary Figure S3E). As expected, RT-PCR showed that psyllium fiber treatment significantly reduced the expression of pro-inflammatory cytokines in liver MNCs (Figure 5E). Therefore, our findings demonstrate that psyllium fiber treatment effectively attenuates alcohol-induced hepatic inflammation.

### 3.6 Psyllium fiber improves hangover symptoms with reduced alcohol metabolites

**FIGURE 6:**
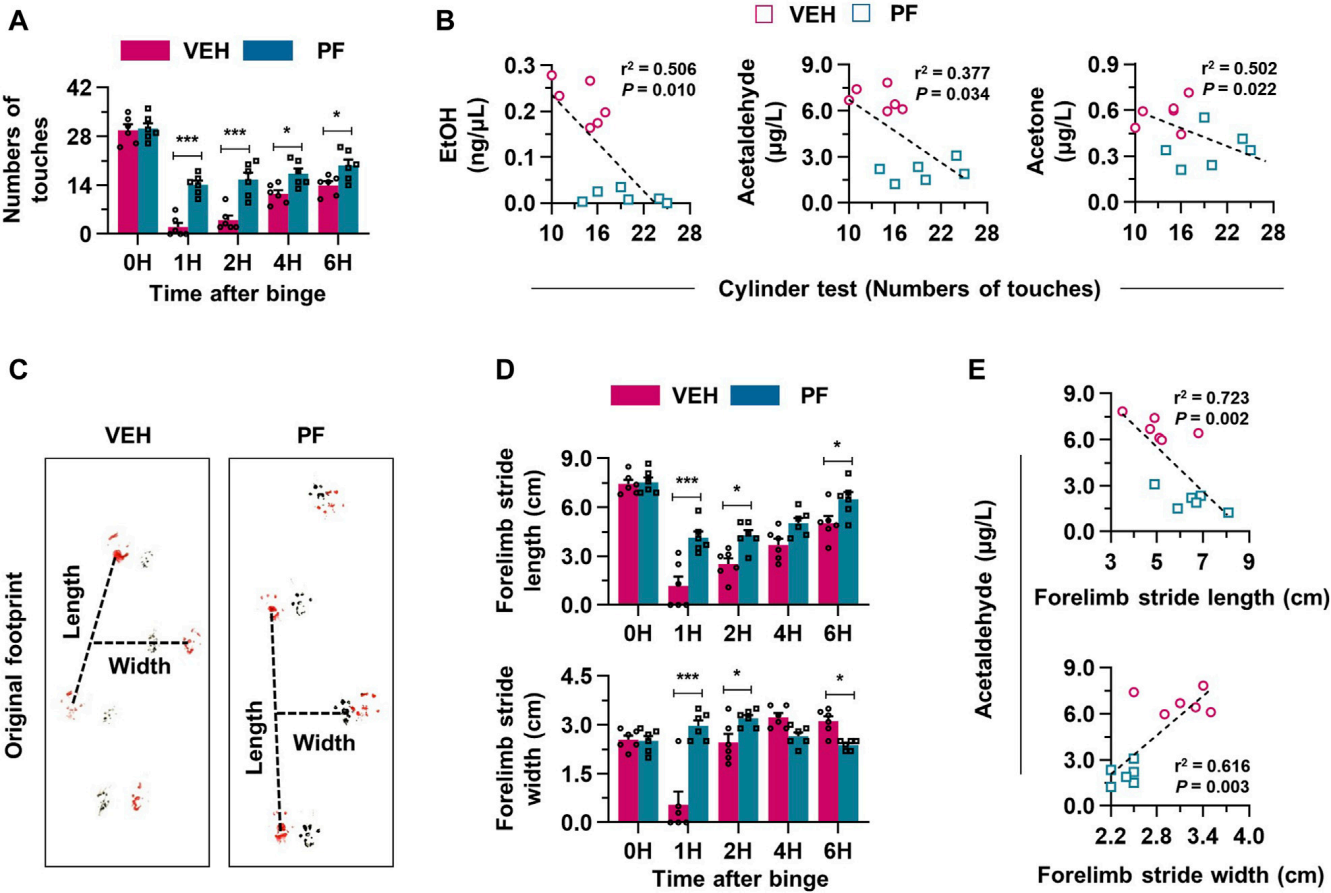
Psyllium fiber effectively improves hangover symptoms with decreased alcohol metabolites. **(A)** Psyllium fiber (PF) increased the number of touches in the cylinder test at different time points (n = 6/group). **(B)** Correlation analysis was performed among alcohol metabolites and cylinder tests in vehicle (VEH) or PF-treated groups (n = 6/group). **(C)** Representative pictures of the footprint test taken 6 h after binge drinking. **(D)** Forelimb stride length and width were compared at the different time points after binge drinking (n = 6/group). **(E)** Correlative analysis between forelimb stride tests and acetaldehyde levels (n = 6/group). Data are presented as mean ± SEM. *p < 0.05, **p < 0.01, ***p < 0.001.

The behavioral effects of psyllium fiber on improving hangover symptoms following binge drinking were analyzed through a series of motor function tests. Supplementary videos show representative cylinder test performances of the normal mice, the binge group treated with vehicle, and the binge group treated with psyllium fiber, respectively (Supplementary Videos S1–S3). Psyllium fiber treatment significantly increased the number of touches in the cylinder test following alcohol consumption, suggesting an improvement in motor coordination and balance in treated mice (Figure 6A). Correlation analyses demonstrated a remarkable relationship in the psyllium fiber group, where an increase in the number of touches in the cylinder test was associated with a decrease in alcohol metabolites, suggesting that the psyllium fiber may enhance recovery from motor impairment following binge drinking (Figure 6B). The footprint test conducted 6 h after alcohol consumption indicated that psyllium fiber-treated mice exhibited improved motor function, characterized by an increase in stride length and a decrease in stride width, reflecting enhanced gait stability (Figure 6C). In the comparison of forelimb and hindlimb strides, the psyllium fiber group displayed an increase in stride length and a decrease in stride width (Figure 6D; Supplementary Figure S4A). In the psyllium fiber group, correlation analysis of forelimb and hindlimb footprint tests showed that as alcohol metabolites decreased, stride length increased and stride width decreased, suggesting an improvement in gait stability and a reduction in staggered walking commonly associated with hangover symptoms (Figure 6E; Supplementary Figures S4B, C). In summary, psyllium fiber alleviates hangover symptoms induced by binge drinking, as evidenced by improved motor functions and reduced alcohol metabolites.

### 3.7 Psyllium fiber suppresses the alcohol absorption into the cells

**FIGURE 7:**
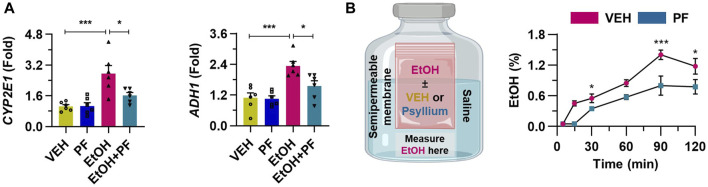
Psyllium fiber inhibits the alcohol absorption into the cells. **(A)** qRT-PCR of *CYP2E1* and *ADH1* in cultured HepG2 cells treated with 100 mM ethanol (EtOH) or 1 mM psyllium fiber (PF) for 6 h (n = 6/group). **(B)** Ethanol concentration was consecutively measured using a semipermeable membrane in samples treated with vehicle (VEH) or 10 mM psyllium fiber (PF) (n = 4/group). Data are presented as mean ± SEM. *p < 0.05, **p < 0.01, ***p < 0.001.

To further demonstrate the hypothesis that psyllium fiber holds alcohol and thereby inhibits its absorption into cells, additional *in vitro* experiments were conducted. When HepG2 cells were treated with ethanol, there was a marked increase in the mRNA levels of CYP2E1 and ADH1, which are essential enzymes in alcohol metabolism. In contrast, due to the inhibitory effect of psyllium fiber on ethanol absorption across the intestinal wall, a noticeable decrease in the expression of these metabolic enzymes was observed (Figure 7A). In the semipermeable membrane, experiment, the treatment with psyllium fiber significantly suppressed the movement of alcohol through the membrane (Figure 7B). This indicates that psyllium fiber may hold onto alcohol, preventing its passage through the membrane, which could imply the ability of psyllium fiber to inhibit the absorption of alcohol in the gastrointestinal tract.

## 4 Discussion

In our study, we provide several lines of evidences that psyllium fiber decreases intestinal metabolism of alcohol after acute drink of alcohol and relieves hangover symptoms (Graphical Abstract). The experimental data using mice suggested that binge drinking upregulates expressions of enzymes including CYP2E1 and ADH within the wall of jejunum and administration of psyllium fiber reduces the expressions. Moreover, impaired motor function of mice with binge drinking dramatically recovered with additional consumption of psyllium fiber with alcohol regarding to the data in this study including the gait dynamic of mice (Kale et al., 2004; Hansen and Pulst, 2013). We also revealed the hepatoprotective effect of psyllium fiber against acute inflammation induced by alcohol drinking using qRT-PCR, flow cytometry, and immunofluorescence staining. In the *in vitro* experiment, we examined the protective mechanisms of psyllium husk fiber on the gut and liver when exposed to alcohol-induced damage. This investigation involved treating HepG2 cell lines and employing semipermeable osmosis membranes to elucidate the details of the process.

A recent interesting study introduced the concept of “intestinal drinking” and it refers to the continue absorption of alcohol in the gut barrier until the defecation, the final release of the fecal materials. The study found that acetaldehyde levels in the blood peaked after binge drinking, and consecutive defecation significantly downregulated the concentration. Furthermore, the study revealed that neutrophil count in the blood and hangover related symptoms such as nausea, headache, and fatigue gradually decreased with the defecation in the next morning of binge drinking. The study emphasized that termination of alcohol absorption within the gastrointestinal wall would relieve hangover related factors (Ryu et al., 2023).

Ethanol is metabolized not only in the liver, but also in the gastrointestinal wall and the fact suggest the gut barrier as the first gatekeeper of alcohol consumption in human body. Ethanol could be metabolized with mucosal cell by ADH and microsomal ethanol oxidizing system (Seitz et al., 1994). A study concluded that ADH and ALDH isozymes are differentially expressed in the jejunum and the result suggest that the small bowel could be the first pass metabolism of alcohol and cytotoxic acetaldehyde would play a pivotal role in gastrointestinal tract (Chiang et al., 2009). Furthermore, gut-liver crosstalk caused by increased gut permeability in alcohol associated liver disease, and inflammatory response induced by upregulated expression of TNF-α, IL-1β and IL-6 in distal ileum with chronic exposure to alcohol support the importance of alcohol metabolism of the intestinal wall (Shim and Jeong, 2020). Collectively, this study aims to highlight the potential efficacy of medical interventions targeting the gastrointestinal wall in alleviating hangover symptoms following binge drinking. We propose the concept of faster alcohol deprivation in the gut with psyllium fiber as a promising approach to mitigate hangover severity.

Psyllium fiber is one of the fiber laxatives that work by increasing the weight and hydrophilic characteristics of stool, thereby softening the consistency of the stool. It is a soluble fiber that blends with water and forms a gelatin-like substance in gastrointestinal tract. Soluble fibers including psyllium fiber is one of the safe and well-tolerated laxatives of which adverse events are rarely occurred with mild abdominal distension (Jensen et al., 2013; Singh et al., 2021). A review article addressed that psyllium as level II evidence and grade B recommendation as a laxatives (Rao and Brenner, 2021). This bulking agent that is a non-absorbable in gastrointestinal tract, absorbs water and enhances intestinal peristalsis deemed safe even during pregnancy (Clementi and Weber-Schöndorfer, 2015).

Previous studies examining hangover improvement have highlighted the increased activities of alcohol-metabolizing enzymes with various medical interventions. A recent study addressed the plant-base extract mixture (M. crystallinum, P. lobata flower, and A. indica) could decrease blood acetaldehyde level and hangover symptoms such as thirst and tiredness, and mechanistic study revealed that blood ADH and ALDH level significantly increased right after the alcohol consumption with the treatment (Jung et al., 2023). Several studies suggest that herbal medicines including Camellia sinensis, Houttuynia cordata, *Nelumbo nucifera* G., and Opuntia ficus indica suggest that activation of ADH and ALDH could be the key mechanism of the hangover relief (Moslemi et al., 2023). Furthermore, fermented smilax china root extract, and fermented persimmon juice also emphasized the importance of increased expression of alcohol metabolizing enzymes with medical intervention after acute alcohol consumption (Zhou et al., 2019; Boby et al., 2021). Nevertheless, our study aims the downregulation of the ethanol metabolizing enzymes, interrupting the absorption of alcohol through the gastrointestinal wall and we analyzed the consequences of the psyllium fiber administration, a bulk forming laxatives.

Recent studies highlight the significance of the gut-liver axis in alcohol-associated liver disease, underscoring the potential of psyllium fiber intervention to not only alleviate hangover symptoms but also mitigate the inflammatory insults to the liver induced by excessive alcohol metabolism. Binge drinking not only leads to risky behavioral habits, impaired cardiovascular system, and gut inflammation, but also induces increased intestinal permeability, upregulated serum nitric oxide, excess of lipid peroxidation products, and sinusoidal endothelia cell dysfunction, thereby results in worsened liver injury and increased portal pressure (Mathurin and Deltenre, 2009). In this research, we investigated the protective effect of psyllium fiber against absorption of alcohol from the gastrointestinal tract, which might reduce the ethanol exposure to the liver via portal vein or systemic circulation. By downregulating “intestinal drinking,” psyllium fiber may not only alleviate hangover symptoms but also exert a hepatoprotective effect against acute liver inflammation. This was evidenced by histologic findings and flow cytometry analysis, which demonstrated a reduction in neutrophil infiltration with the medical intervention. Also, we conducted *in vitro* experiment that could explain the liver protection of psyllium fiber using HepG2 cell line and expression of alcohol metabolizing enzymes such as CYP2E1 and ADH.

Our findings also emphasized the osmotic effect of psyllium fiber, that result in retention of water in the gastrointestinal tract, and it could also hold alcohol that has hydrophilic characteristics. We specified this mechanism utilizing semipermeable membrane experiment. Ethanol plus vehicle or ethanol plus psyllium fiber were inserted in the semipermeable pocket, and the pocket was soaked in the saline bottle. Psyllium fiber effectively held water and additional ethanol, thereby inhibiting the leakage of ethanol from inside of pocket to the saline bottle (Figure 7B). The experimental data suggest that administered psyllium fiber could hold water and hydrophilic ethanol by composing gelled structure throughout the gastrointestinal tract. This experimental mechanism is consistent with the mode of action as a laxatives of psyllium, which is explained by water retaining in the small intestine, and increasing water flow into the distal ileum and ascending colon, resulting increase in the fluidity of the colonic materials (Jalanka et al., 2019).

There may be concerns that alleviating hangover symptoms could potentially exacerbate alcoholism by encouraging further alcohol consumption. However, existing literature suggests that hangover, often accompanied by depressed mood, is a part of the vicious cycle of alcoholism (Wiese et al., 2000; Penning et al., 2010). Thus, relieving hangover symptoms could potentially disrupt this cycle. Moreover, research indicates that the elimination of alcohol and acetaldehyde through defecation may offer a solution not only for alleviating hangover symptoms but also for addressing alcohol abuse (Ryu et al., 2023).

The present study has several limitations that warrant acknowledgment. First, we could not conduct obtain human blood samples after binge drinking. For the further investigation, human cohort study with or without psyllium fiber after alcohol consumption would be required. Next, the effect of psyllium fiber was limited to the gut protection and hepatoprotective effect after binge drinking. In future experiments, it would be beneficial to explore the potential hangover protection mechanisms involving other organs, such as the brain or bone marrow, which are known to interact with the liver during acute alcohol-induced damage.

Based on the extensive experimental data gathered in this study, we propose that the administration of psyllium husk could serve as an effective treatment option for alleviating hangover symptoms and providing additional protection to the gastrointestinal tract and liver against acute alcohol-induced damage. Further supplementary experiments are warranted to explore the potential application of psyllium fiber as a safe and effective medical intervention for hangovers in real-world scenarios.

## Data Availability

The original contributions presented in the study are included in the article/Supplementary Material, further inquiries can be directed to the corresponding authors.
