# Supplementary material for: Psyllium fiber improves hangovers and inflammatory liver injury by inhibiting intestinal drinking
Source: Front Pharmacol. 2024 Jun 28;15:1378653. doi: 10.3389/fphar.2024.1378653 (PMC11239518; doi:10.3389/fphar.2024.1378653)
Supplement: Supplementary file 2 [file Table1.DOCX]

**Supplementary Table 1. Primer sequences for qPCR.**

| **Genes** | **Forward** | **Reverse** |
| --- | --- | --- |
| ***18S*** | ACAGGATTGACAGATTGATAGC | GCCAGAGTCTCGTTCGTTA |
| ***Cyp2e1*** | CGTTGCCTTGCTTGTCTGGA | AAGAAAGGAATTGGGAAAGGTCC |
| ***Adh1*** | GCAAAGCTGCGGTGCTATG | TCACACAAGTCACCCCTTCTC |
| ***Tnf*** | AAGCCTGTAGCCCACGTCGTA | AAGGTACAACCCATCGGCTGG |
| ***Il1b*** | GCCCATCCTCTGTGACTCAT | AGGCCACAGGTATTTTGTCG |
| ***Cxcl1*** | ACTGCACCCAAACCGAAGTC | TGGGGACACCTTTTAGCATCTT |
| ***ACTB*** | AGCGAGCATCCCCCAAAGTT | GGGCACGAAGGCTCATCATT |
| ***CYP2E1*** | GCAAGAGATGCCCTACATGGA | GGGCACGAGGGTGATGAA |
| ***ADH*** | AGTCATCCCACTCGCTATTCC | GTCCCCTGAGGATTGCTTACA |
