# Supplementary material for: Psyllium fiber improves hangovers and inflammatory liver injury by inhibiting intestinal drinking
Source: Front Pharmacol. 2024 Jun 28;15:1378653. doi: 10.3389/fphar.2024.1378653 (PMC11239518; doi:10.3389/fphar.2024.1378653)
Supplement: Supplementary file 3 [file Presentation1.PPTX]

## Slide 1
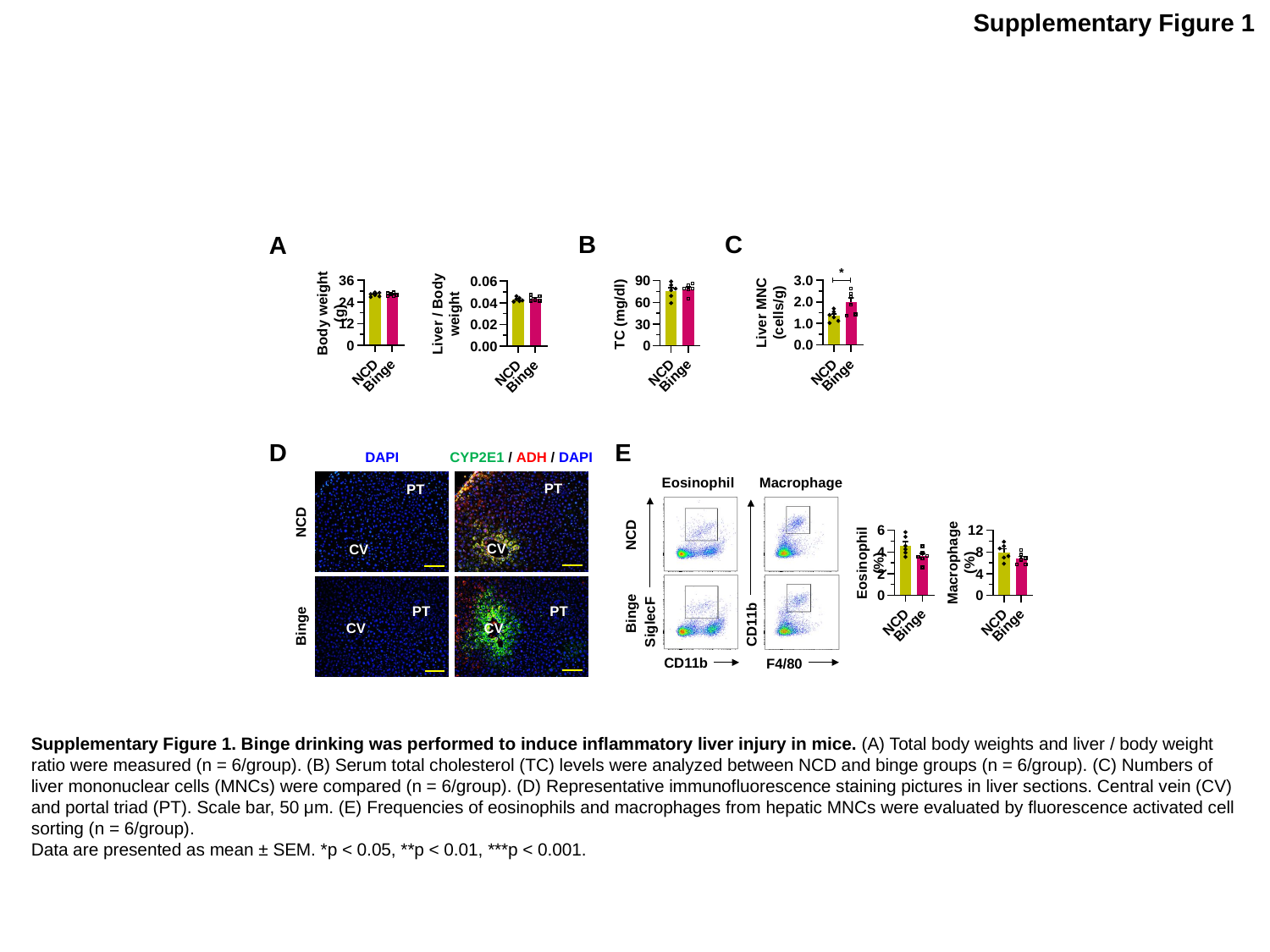

Supplementary Figure 1
B
C
A
Body weight (g)
*
Liver MNC
(cells/g)
Liver / Body weight
TC (mg/dl)
D
E
DAPI
CYP2E1 / ADH / DAPI
Macrophage
Eosinophil
PT
PT
Macrophage (%)
Eosinophil (%)
NCD
NCD
CV
CV
PT
PT
Binge
SiglecF
CD11b
Binge
CV
CV
CD11b
F4/80
Supplementary Figure 1. Binge drinking was performed to induce inflammatory liver injury in mice. (A) Total body weights and liver / body weight ratio were measured (n = 6/group). (B) Serum total cholesterol (TC) levels were analyzed between NCD and binge groups (n = 6/group). (C) Numbers of liver mononuclear cells (MNCs) were compared (n = 6/group). (D) Representative immunofluorescence staining pictures in liver sections. Central vein (CV) and portal triad (PT). Scale bar, 50 μm. (E) Frequencies of eosinophils and macrophages from hepatic MNCs were evaluated by fluorescence activated cell sorting (n = 6/group).
Data are presented as mean ± SEM. *p < 0.05, **p < 0.01, ***p < 0.001.

## Slide 2
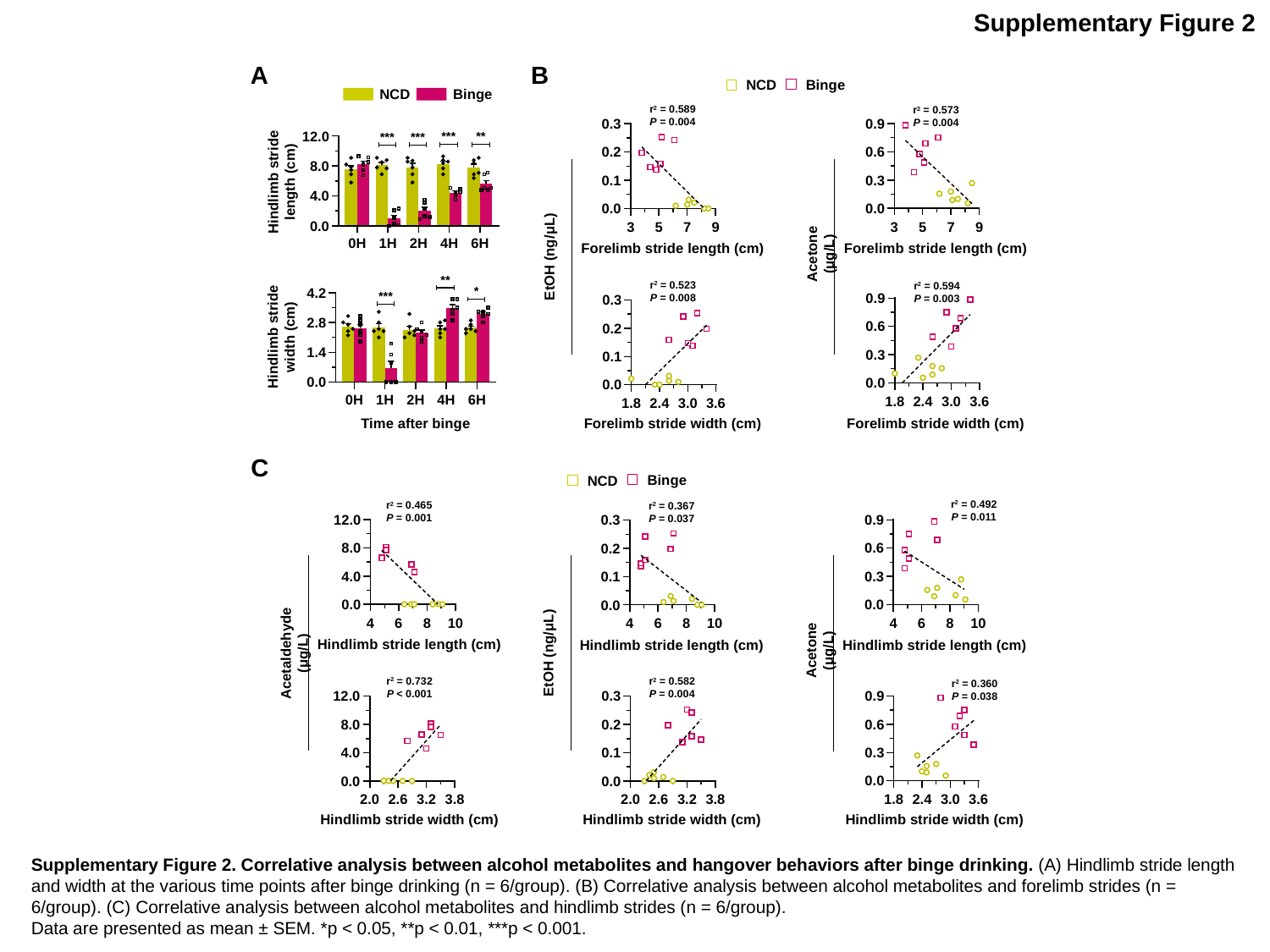

Supplementary Figure 2
A
B
Binge
NCD
Binge
NCD
r2 = 0.589
P = 0.004
r2 = 0.573
P = 0.004
***
**
***
***
Hindlimb stride length (cm)
Forelimb stride length (cm)
Forelimb stride length (cm)
Acetone (μg/L)
EtOH (ng/μL)
**
*
r2 = 0.523
P = 0.008
r2 = 0.594
P = 0.003
***
Hindlimb stride width (cm)
Forelimb stride width (cm)
Forelimb stride width (cm)
Time after binge
C
Binge
NCD
r2 = 0.492
P = 0.011
r2 = 0.465
P = 0.001
r2 = 0.367
P = 0.037
Hindlimb stride length (cm)
Hindlimb stride length (cm)
Hindlimb stride length (cm)
Acetone (μg/L)
EtOH (ng/μL)
Acetaldehyde (μg/L)
r2 = 0.732
P < 0.001
r2 = 0.582
P = 0.004
r2 = 0.360
P = 0.038
Hindlimb stride width (cm)
Hindlimb stride width (cm)
Hindlimb stride width (cm)
Supplementary Figure 2. Correlative analysis between alcohol metabolites and hangover behaviors after binge drinking. (A) Hindlimb stride length and width at the various time points after binge drinking (n = 6/group). (B) Correlative analysis between alcohol metabolites and forelimb strides (n = 6/group). (C) Correlative analysis between alcohol metabolites and hindlimb strides (n = 6/group).
Data are presented as mean ± SEM. *p < 0.05, **p < 0.01, ***p < 0.001.

## Slide 3
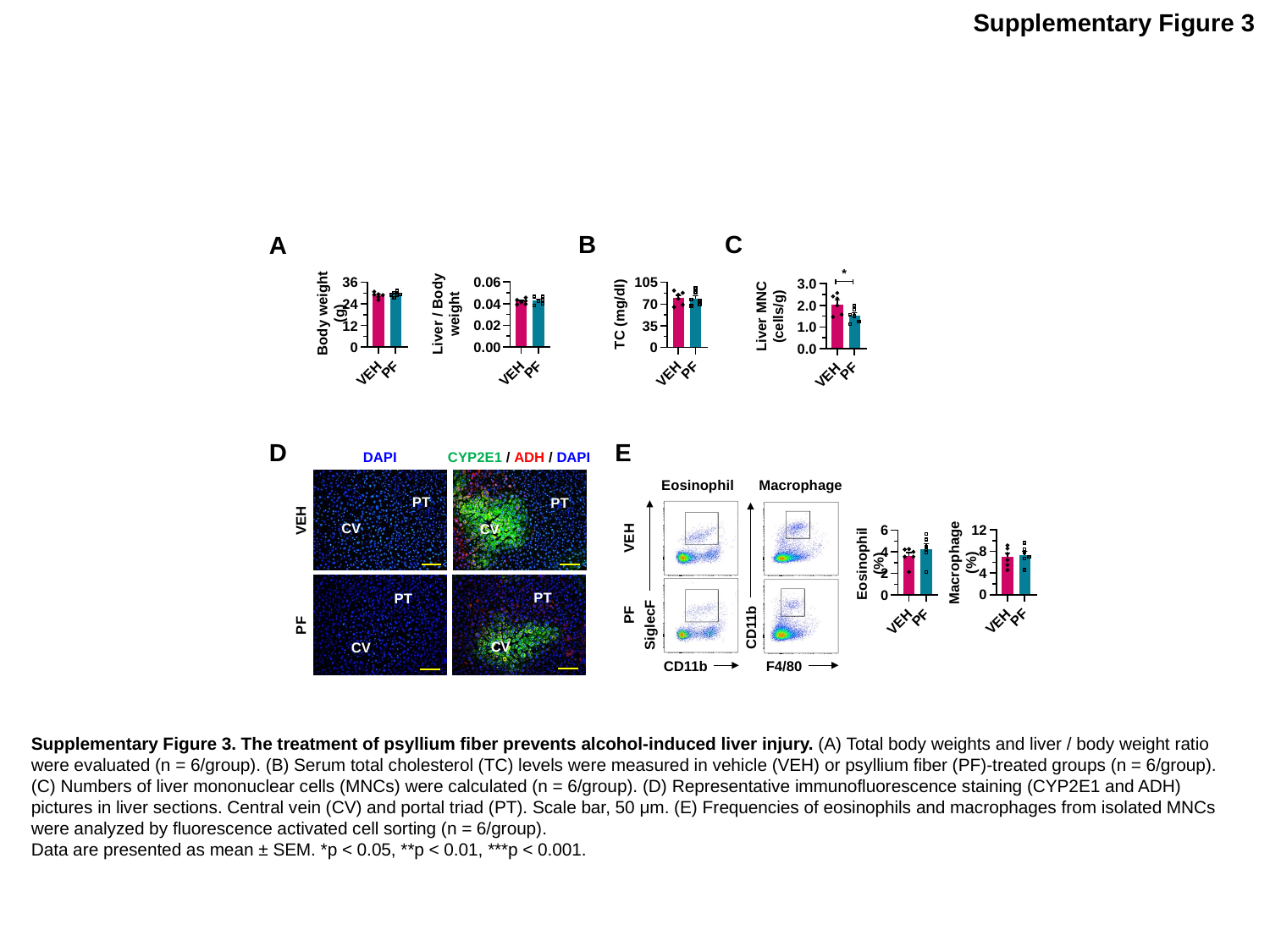

Supplementary Figure 3
B
C
A
Body weight (g)
TC (mg/dl)
*
Liver / Body weight
Liver MNC
(cells/g)
D
E
DAPI
CYP2E1 / ADH / DAPI
Macrophage
Eosinophil
PT
PT
Macrophage (%)
Eosinophil (%)
VEH
CV
CV
VEH
PT
PT
PF
SiglecF
PF
CD11b
CV
CV
CD11b
F4/80
Supplementary Figure 3. The treatment of psyllium fiber prevents alcohol-induced liver injury. (A) Total body weights and liver / body weight ratio were evaluated (n = 6/group). (B) Serum total cholesterol (TC) levels were measured in vehicle (VEH) or psyllium fiber (PF)-treated groups (n = 6/group). (C) Numbers of liver mononuclear cells (MNCs) were calculated (n = 6/group). (D) Representative immunofluorescence staining (CYP2E1 and ADH) pictures in liver sections. Central vein (CV) and portal triad (PT). Scale bar, 50 μm. (E) Frequencies of eosinophils and macrophages from isolated MNCs were analyzed by fluorescence activated cell sorting (n = 6/group).
Data are presented as mean ± SEM. *p < 0.05, **p < 0.01, ***p < 0.001.

## Slide 4
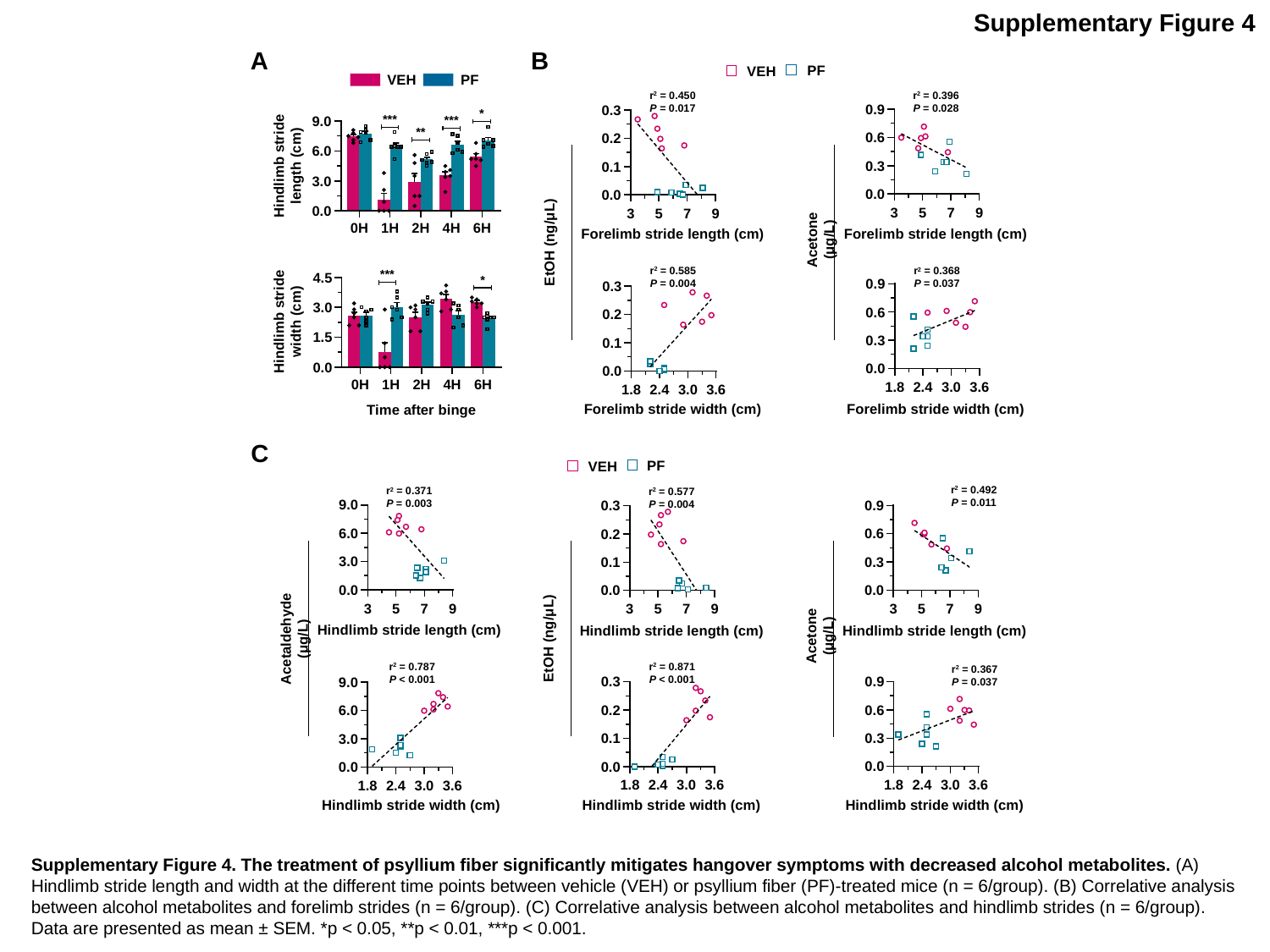

Supplementary Figure 4
A
B
PF
VEH
PF
VEH
r2 = 0.450
P = 0.017
r2 = 0.396
P = 0.028
*
***
***
**
Hindlimb stride length (cm)
Forelimb stride length (cm)
Forelimb stride length (cm)
Acetone (μg/L)
EtOH (ng/μL)
***
r2 = 0.585
P = 0.004
r2 = 0.368
P = 0.037
*
Hindlimb stride width (cm)
Forelimb stride width (cm)
Forelimb stride width (cm)
Time after binge
C
PF
VEH
r2 = 0.492
P = 0.011
r2 = 0.371
P = 0.003
r2 = 0.577
P = 0.004
Hindlimb stride length (cm)
Hindlimb stride length (cm)
Hindlimb stride length (cm)
Acetone (μg/L)
EtOH (ng/μL)
Acetaldehyde (μg/L)
r2 = 0.787
P < 0.001
r2 = 0.871
P < 0.001
r2 = 0.367
P = 0.037
Hindlimb stride width (cm)
Hindlimb stride width (cm)
Hindlimb stride width (cm)
Supplementary Figure 4. The treatment of psyllium fiber significantly mitigates hangover symptoms with decreased alcohol metabolites. (A) Hindlimb stride length and width at the different time points between vehicle (VEH) or psyllium fiber (PF)-treated mice (n = 6/group). (B) Correlative analysis between alcohol metabolites and forelimb strides (n = 6/group). (C) Correlative analysis between alcohol metabolites and hindlimb strides (n = 6/group).
Data are presented as mean ± SEM. *p < 0.05, **p < 0.01, ***p < 0.001.
